# Radical resection of locally advanced and recurrent colorectal carcinoma involving major nerve resection: a systematic review of surgical, oncological and functional outcomes

**DOI:** 10.1007/s00384-024-04707-7

**Published:** 2024-08-20

**Authors:** Justin A. Hawke, Samantha Regora, Amrish Rajkomar, Alexander Heriot, Helen Mohan, Satish Warrier

**Affiliations:** 1 Division of Cancer Surgery, Peter MacCallum Cancer Centre, Victorian Comprehensive Cancer Centre, Melbourne, VIC Australia; 2 The University of Melbourne, Parkville, VIC Australia; 3 Epworth Healthcare, Richmond, VIC Australia; 4 Monash University, Clayton, VIC Australia

**Keywords:** Colorectal neoplasms, Colorectal surgery, Pelvic exenteration, Peripheral nerves

## Abstract

**Background:**

The aim of this study was to explore the surgical, oncological and quality of life outcomes in the setting of radical resection of colorectal carcinoma involving major nerve resection.

**Methods:**

A systematic review of the literature was registered with the International Prospective Register for Systematic Reviews (PROSPERO) and performed following Preferred Reporting Items for Systematic Reviews and Meta-Analyses (PRISMA) guidelines to identify papers relating to outcomes in radical resection of colorectal cancer where major nerve resection was undertaken. Papers were identified from OVID Medline, EMBASE Classic and Web of Science encompassing all publications in English from January 2010 to June 2023. A total of 1357 nonduplicate studies were identified and screened for relevance, with six studies included in the final review.

**Results:**

A total of 354 major nerve resections were undertaken across the six included studies. Overall postoperative morbidity was reported at rates of up to 82%. Two studies considered nerve-resection-specific oncological outcomes, with complete pathological resection achieved at rates comparable to the wider pelvic exenteration cohort (65–68%) and without any overall survival disadvantage being conveyed by major nerve resection (*p* = 0.78). Two studies considered functional outcomes and noted a transient decrease in physical quality of life over the first 6 months postoperatively (*p* = 0.041) with significant loss to follow-up. One study considered postoperative pain in nerve resection and noted no significant increase in patient-reported pain scores associated with nerve resection (*p* = 0.184–0.618).

**Conclusions:**

Major nerve resections in locally advanced and recurrent colorectal cancer remain understudied but with encouraging initial oncological and functional outcomes. Multicentre collaborative prospective reviews are needed to better elucidate contributors to postoperative morbidity and functional deficits and further establish interventions to ameliorate them.

## Introduction

Surgical resection with curative intent for locally advanced colon and rectal cancer has traditionally involved multivisceral resection, an approach that utilises *en bloc* resection of the tumour and adjacently involved organs, viscera and neurovasculature [1, 2]. Curative intent surgery encompasses resecting all involved structures and organs to achieve a pathologically clear margin whilst navigating an appropriate risk of morbidity and mortality and achieving acceptable postoperative quality of life [1, 3]. This approach requires appropriate patient selection, robust perioperative supports and multidisciplinary postoperative management to achieve optimal postoperative outcomes. Postoperatively, patients require stoma management counselling and titration of multimodal analgesic regimes and undergo lengthy recuperative times with significant rehabilitation requirements [4–7]. Locally recurrent colorectal malignancies are managed in a similar fashion in undertaking radical resection of the tumour and its attached structures in an *en bloc* fashion [3].

Locally advanced and recurrent colorectal cancer involving major pelvic nerves and aortoiliac axis vasculature was historically considered inoperable in the setting of an unacceptably high associated risk of injury and poor postoperative quality of life [8]. In the last decade, novel surgical techniques involving higher and wider pelvic exenterations and/or radical en bloc resection of these tumours have been developed [9]. These approaches are technically feasible in at least half of clinical presentations and are now recognised as the optimal surgical approach to offer patients with locally recurrent colorectal cancer their best chance at long-term survival [10]. Where radical resection is undertaken, clear pathological (R0) margins remain critical to long-term survival [8, 9, 11–14].

Emerging data where radical resection of nerves and vessels is undertaken in the curative resection of locally advanced or recurrent rectal cancer has been oncologically promising. Brown et al. noted 5-year survival rates of 55% and a median survival of 41 months in their retrospective review of 39 patients undergoing curative resection for colorectal cancer with nerve involvement when R0 margins were achieved [15]. Beyond the critical importance of complete macroscopic resection, strict preoperative patient selection criteria, multidisciplinary review, increasing familiarity and volume of involved surgical techniques were noted to be significant contributors to achieved outcomes [15]. Previous prospective studies have also noted that overall postoperative rates of complications where surgery involved nerve resection were not significantly different from rates in current pelvic exenteration surgeries without nerve resection [16].

Patients undergoing nerve resection can experience functional sequelae including but not limited to foot drop. In such patients, ankle foot orthosis is required to mobilise, with independent mobilisation representing a key functional target outcome [15]. Quality of life outcomes in radical colorectal cancer resections involving major pelvic nerve resection remains understudied and has not been examined systematically within the literature, with individual studies considering this postoperative domain hampered by significant loss-to-follow-up rates [15].

The surgical, oncological and functional outcomes of radical resection involving major pelvic nerves have not previously been investigated in a systematic fashion. This study aimed to undertake a systematic review of existing literature on surgical, oncological and functional outcomes in radical resection of locally advanced and recurrent colorectal malignancy involving nerve resection.

## Methods

A systematic review was registered with PROSPERO international prospective register of systematic reviews (ID CRD42023447615). Following the Preferred Reporting Items for Systematic Reviews and Meta-Analyses (PRISMA) guidelines, a literature search was conducted in June 2023 with specific regards to the surgical, oncological, functional and pain outcomes following locally advanced and recurrent colorectal carcinoma radical resection with nerve resection. The search encompassed OVID Medline (including Embase Classic), as well as Web of Science, for papers from January 2010 to June 2023 written in English. Key search words included ‘colorectal’, ‘pelvic neoplasms’, ‘radical resection’ and ‘pelvic nerves’. The full search strategy is attached and identified 1357 papers in total after the exclusion of duplicate studies (Appendix 1).

Rayyan systematic review software was used to collate and review abstracts after exclusion of duplicate studies [17]. Independent, blinded title and abstract screening of the initial pool of papers was completed in duplicate (by JH and SR) following the pre-determined inclusion and exclusion criteria (Table 1). Conflicts between reviewers were resolved by consensus and/or the input of a third reviewer (HM) where required. Full-text reviews were subsequently retrieved and reviewed to determine final inclusion eligibility. Reference lists of articles that were deemed to be included were further assessed for manual extraction of relevant further articles.

**Table 1:** Inclusion and exclusion criteria

	Inclusion criteria	Exclusion criteria
Study design	- Randomised controlled trials, prospective studies, retrospective studies	- Other surgeries/tumour types (non-colorectal), case reports
Population	- Patients of any age with locally advanced or locally recurrent colorectal malignancy - Surgical excision has been undertaken involving major nerve resection (sciatic, obturator, femoral or sacral nerve roots) - Curative intent surgery	- Papers not assessing a cohort of predominantly colorectal malignancy radical resection - Studies where patients have not undergone surgical management (i.e. chemotherapy, radiotherapy or palliative monotherapies) - Palliative intent surgery
Intervention	- Radical resection of locally advanced and locally recurrent colorectal cancer involving major nerve resection	N/A
Comparison	- Comparison to radical resection not involving major nerve resection	N/A
Outcome	- Operative outcomes, oncological outcomes, functional outcomes, pain	N/A

### Data extraction

Data was extracted for study design, country, recruitment period, participants, surgical interventions, resection intent and outcome. Data was further collected on surgical outcomes including median age, duration, primary/recurrent tumour, nerves resected, length of stay, blood loss and complications. Oncological outcomes extracted included R0 margins, overall survival and recurrence-free survival. Functional outcomes extracted data included mobilisation and neuropathic pain data, FACT-C quality of life score differences and 6-min walk test results. Pain-related outcomes included preoperative pain, peroperative opioids, postoperative pain and opioids further separated by specific nerve excision.

### Assessment and methodological quality and risk of *bias*

The methodological quality and risk of bias for each study were analysed using the Newcastle–Ottawa Scale risk of bias assessment tool (Appendix 2).

## Results

**Fig. 1:**
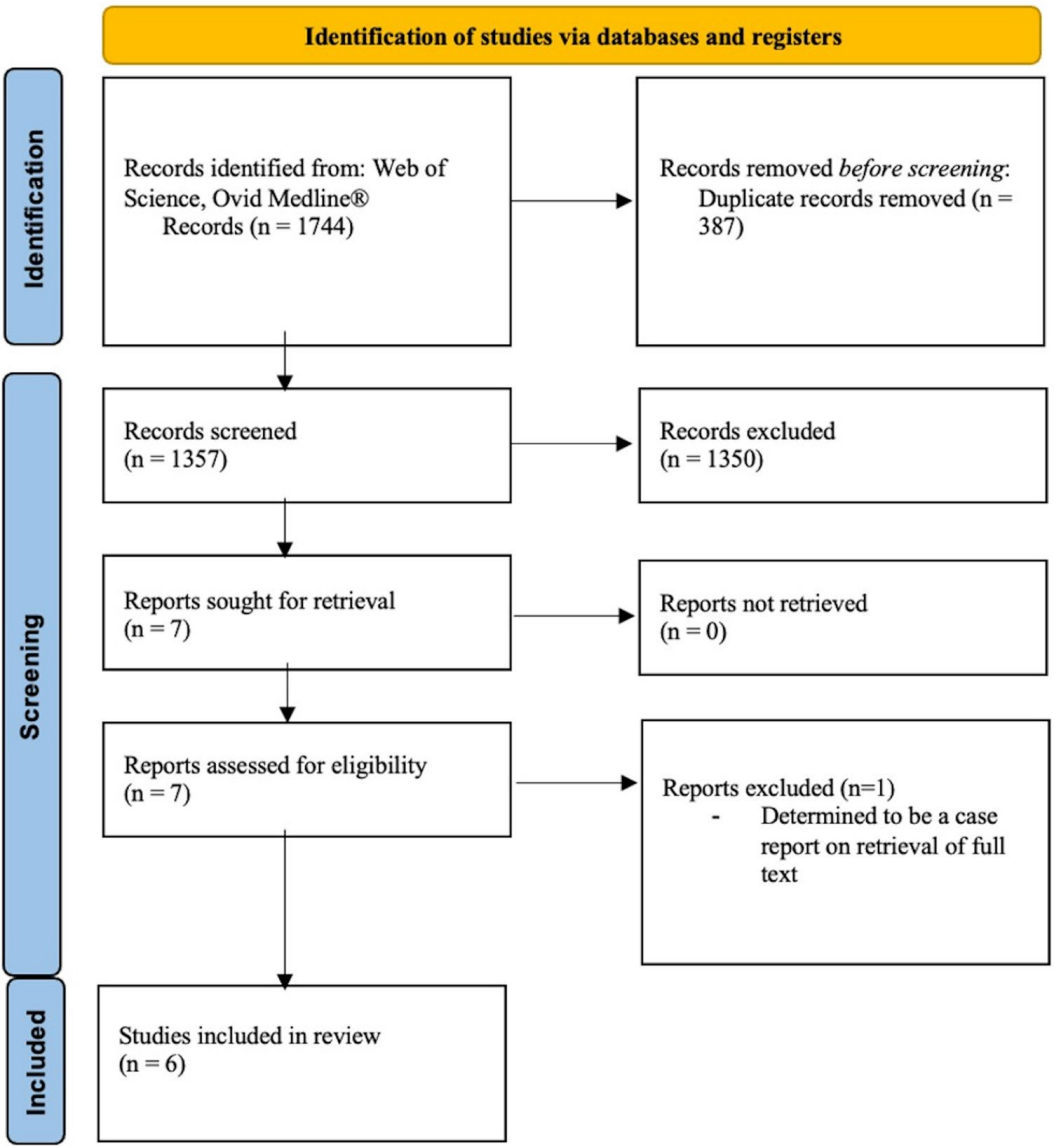
Preferred Reporting Items for Systematic Reviews and Meta-Analyses (PRISMA) flow diagram outlining the identification and screening of included articles

The initial search yielded 1357 non-duplicated studies for abstract screening. Seven journal articles were subsequently assessed in full-text form for eligibility with a final six papers included in the final analysis as outlined within the included PRISMA flow diagram (Fig. 1). A broad search strategy combined with inclusion criteria focused specifically on colorectal cancer resulted in a large proportion of excluded papers as noted within the PRISMA diagram (Fig. 1). Data extracted from these papers was subsequently summarised and is found in Tables 2, 3, 4, 5 and 6. The demographics of included studies, surgical, oncological and functional outcomes following radical resection of locally advanced and recurrent colorectal carcinoma involving major nerve resections are explored below (Appendix 3).

**Table 2:** Characteristics of included studies

Study	Country	Recruitment period	Study design	Number of eligible participants	Surgical intervention	Resection intent	Outcome
Shaikh et al. [9]	England	2011–2013	Retrospective cohort study	6 (3 with major nerve resection)	Pelvic exenteration with en bloc ELSiE and sciatic nerve resection	Curative	R0 oncological margins achieved in all cases
Solomon et al. [16]	Australia	1994–2014	Retrospective cohort study	200 patients (27 with major nerve resection)	Pelvic exenteration with en bloc lateral compartment resection involving major nerve resection	183 curative, 17 palliative	No overall survival disadvantage (*p* = 0.78) or disease-free survival disadvantage (*p* = 0.773) in univariate analysis from nerve resection
Lim et al. [7]	Australia	2013–2014	Retrospective cohort study	99 patients (61 with major nerve resection)	Pelvic exenteration with en bloc lateral compartment resection involving major nerve resection	Curative	Major nerve excision (sciatic, gluteal, obturator, femoral) not significantly associated with worse postoperative pain as measured by VNRS
Makker et al. [18]	Australia	2017–2020	Retrospective cohort study	135 patients (45 underwent major nerve resection)	Pelvic exenteration with en bloc lateral compartment resection involving major nerve resection	Curative	No significant difference in 6-min walk test outcomes between patients undergoing exenteration with and without major nerve resection at baseline, at 10 days and at discharge
Brown et al. [15]	Australia	1994–2018	Retrospective cohort study	68 patients (40 colorectal cancers)	Pelvic exenteration with en bloc lateral compartment resection involving major nerve resection	Curative (39), palliative (1)	R0 resection rate of 68% in colorectal cancer Median overall survival of 41 months in colorectal cancer patients Overall and major complication rates of 63% and 40% Physical quality of life score significant lower six months postoperative (*p* = 0.041), not significantly returned to baseline at 12 months
Sutton et al. [17]	Australia	1994–2020	Retrospective cohort study	716 patients (178 with major nerve resection)	Pelvic exenteration with en bloc lateral compartment resection involving major nerve resection	Curative	Statistically significant association between major nerve resection and subsequent need for surgery for empty pelvis syndrome (16 of 178 patients) (*p* = 0.03)

**Table 3:** Surgical outcomes

Study	Age (median)	Duration (median)	Primary/recurrent tumour	Nerves resected	Length of stay (median)	Blood loss	Complications
Shaikh et al. [9]	55 years	9 h	3 recurrent rectal, 1 primary rectal, 1 recurrent anal, 1 re-recurrent anal	Sciatic (*n* = 3)	21 days (range 20–33 days)	800 mL (median)	66% Clavien-Dindo II (1 bladder leak managed conservatively, 1 pelvic collection treated with antibiotics)
Solomon et al. [16]	60 years	10 h 15 min	100 recurrent rectal, 32 primary rectal, 18 recurrent anal, 1 primary anal, 6 primary colon, 4 recurrent colon, 39 other	Sciatic (*n* = 27), obturator (*n* = 29), femoral (*n* = 2)	25 days (range 5–189)	3.5 L (median)	164 total complications (82%)—minor (I/II) 79.3%, major (III/IV) 27.6%
Lim et al. [7]	57.5 years	Not reported	Primary rectal (*n* = 27), recurrent local (*n* = 25) and other (*n* = 47)	Sciatic (*n* = 14), gluteal (*n* = 23), femoral (*n* = 1), obturator (*n* = 19)	21 days	Not reported	Perineal wound complications (*n* = 19)
Makker et al. [18]	61 years	9.7 h	Primary rectal (*n* = 45 + 36), recurrent rectal (*n* = 48 + 28)	Sacral (*n* = 45 + 30), femoral (*n* = 2 + 1), obturator (*n* = 21 + 20), sciatic (*n* = 22 + 20), gluteal (*n* = 44 + 27)	22 days	Not reported	Total (*n* = 124 + 104) with focus on wound, gastrointestinal, stoma, cardiovascular, sepsis and haemorrhage
Brown et al. [15]	56 years	10.3 h	27 recurrent rectal, 6 re-recurrent rectal, 5 primary rectal, 2 primary colorectal, 1 recurrent colorectal, 1 rectal carcinoid (general outcomes include 8 primary sarcoma, 7 recurrent anal SCC, 6 recurrent sarcoma, 2 recurrent ovarian, 2 recurrent cervical)	Sciatic (complete), 38; sciatic (partial), 26; femoral, 4	27.5 days (range 7 to 196 days)	4.2 L (median)	43 patients total (63%)—34 (50%) Clavien-Dindo I/II, 27 (40%) Clavien-Dindo III/IV, 19 returns to theatre
Sutton et al. [17]	61 years		Locally recurrent (*n* = 45), distant (*n* = 91), local and distant (*n* = 20) and primary (560)	Major nerve (*n* = 178)	22 days (14–34), ICU 3 days (0–5)	1.5 L median (0.9–3)	Overall (*n* = 611 = 85%), Clavien-Dindo, 11% required operative management for complications within 90 days, 53% for EPS and 6% complex fistula

**Table 4:** Postoperative complications

Study	Complications
Shaikh et al. [9]	66% Clavien-Dindo II (1 bladder leak managed conservatively, 1 pelvic collection treated with antibiotics)
Solomon et al. [16]	164 total complications (82%)—minor (I/II) 79.3%, major (III/IV) 27.6% Major complications most commonly included pelvic collection requiring intervention (*n* = 26, 13% of major), urological leak (14 of 102, 13.7%) and wound/flap dehiscence or necrosis (*n* = 12, 6%). Minor complications included pelvic collection not requiring intervention (*n* = 33, 16.5% of minor), atelectasis (*n* = 31, 15.5%) and prolonged ileus (*n* = 29, 14.5%)
Lim et al. [7]	Perineal wound complications (*n* = 19)—given its focus on pain outcomes, this paper does not elaborate further on complications other than to note that the presence of these complications was not associated with higher rates of opioid prescription or dose
Makker et al. [18]	Total 228—most commonly gastrointestinal (*n* = 118), wound (*n* = 96) and stomal (*n* = 97) complications
Brown et al. [15]	With specific regard to nerve resection, 43 patients total (63%)—34 (50%) Clavien-Dindo I/II, 27 (40%) Clavien-Dindo III/IV, 19 returns to theatre (*n* = 8 for perineal wound dehiscence, *n* = 5 for urological reconstruction complications, *n* = 5 for leak or haemorrhage)
Sutton et al. [17]	Overall (*n* = 611 = 85%), Clavien-Dindo, 11% required operative management for complications within 90 days, 53% for EPS and 6% complex fistula

**Table 5:** Oncological outcomes

Study	R0 margins	Overall survival	Recurrence-free survival
Shaikh et al. [9]	100% (*n* = 6)	Not reported	Not reported
Solomon et al. [16]	Not specifically reported for nerve resection subcohort (R0 = 68.9% for general curative exenteration cohort)	No overall survival disadvantage from sciatic nerve excision (*p* = 0.78 for any significance difference)	No disease-free survival disadvantage from sciatic nerve excision (*p* = 0.773 for any significance difference)
Lim et al. [7]	Not specifically reported for nerve resection subcohort or for colorectal cancer specifically	Not reported	Not reported
Makker et al. [18]	Not specifically reported for nerve resection subcohort (R0 = 84.2% for general curative exenteration cohort)	Not reported	Not reported
Brown et al. [15]	R0 65% for overall colorectal cancer and associated with 5-year overall survival advantage (55% vs 23%) (*p* = 0.006) 21 of 38 (68%) for locally recurrent colorectal cancer and associated with 5-year overall survival advantage (*p* = 0.006)	**Primary colorectal** 1 y 92% 3 y 54% 5 y 43% Median 41 months (95% CI (21.9–60.1)) **Recurrent colorectal** 1 y 93% 3 y 52% 5 y 41%	**Overall colorectal cancer** 1 y 76% 3 y 66% 5 y 57% 10 of 36 (28%) patients developed local recurrence R0 resection associated with higher rates of 2-year recurrence-free survival (89% vs 13%, *p* < 0.001)

**Table 6:** Functional outcomes

Study	Complete sciatic nerve resection	Partial sciatic nerve resection	Outcome
Makker et al. [18]	Not assessed in substratified manner	Not assessed in substratified manner	No statistically significant difference in terms of 6-min walk test following exenteration with nerve resection at baseline, 10 days postop or at discharge
Brown et al. [15]	91% foot drop 35% independent mobility 61% independent mobility with aid 4% wheelchair bound 52% neuropathic pain	32% foot drop 59% independent mobility 33% independent mobility with aid 7% wheelchair bound 65% neuropathic pain	Significant FACT-C physical component score decrease at 6 mo (*p* = 0.041), no statistically significant difference at 12mo (*p* = 0.163)

### Demographics of included studies

All articles were examined for country of study, study design, number of eligible participants, surgical intervention, resection intent and outcome studied (Table 2). All six papers included in this systematic review were retrospective cohort studies that examined various outcomes following pelvic exenteration for locally recurrent rectal cancer with partial or full nerve resection. All included papers were from Australian patient cohorts, except Shaikh et al. which considered an English cohort. It should be noted that five of the six included studies originated from cohorts within a single centre (Royal Prince Alfred Hospital in Sydney) and thus the possibility of individual cases crossing over between multiple papers cannot be excluded. Surgical aims of all studies were of predominantly curative intent, with Brown et al. and Solomon et al. combining their patient cohort with a palliative subset of intent as well (although with outcomes separately reported) [15, 16].

### Surgical outcomes

Surgical outcomes were reported across all papers and included the age of patient, population, primary/recurrent tumour, nerves resected, length of hospital stay and total median blood loss (Table 3). Major nerve resections were completed in all examined studies. In many instances, a lack of differentiation between the total pelvic exenterations alone versus subcohorts undergoing nerve resection specifically was noted. Nerves resected included the sciatic, obturator, femoral and gluteal nerves. Brown et al. further identified complete or partial nerve resections performed.

Minimal variation was found in the areas of age and surgery length across all included studies for pelvic exenterations generally, noting that detailed age and surgery length specific to the nerve resected patients were not reported. The median age of patient cohorts included in these studies was 55–61 years and surgical duration averaged 9 to 10.25 h, albeit with some papers not clearly identifying their operative times and all papers failing to distinguish any added operative time secondary to a nerve resection being performed in addition to the radical pelvic exenteration. Reported median length of hospital stay also reported data generalised to pelvic exenterations overall without stratifying for major nerve resection, with minimal difference between the papers—21 to 27.5 days encompassing all studies. A range of data outliers were reported (e.g. 5–189 days reported in Solomon et al., 7–196 days in Brown et al.) [15, 16].

Postoperative complications were reported in all studies and specifically outlined in our analysis (Table 4). Solomon et al. described morbidity rates of 82%, with 50% of their patient cohort suffering sepsis with origins in urological, intrabdominal, wound-related, intravenous-line related and pulmonary but without discerning data specific to nerve resection patients [16]. Shaikh et al. and Brown et al. utilised the Clavien-Dindo scoring system for complications, describing minor morbidities as class I or II and major complications as III or IV [9, 15]. One paper detailed complications specifically to the subcohort of pelvic exenteration surgery with radical nerve resection and reported that return to theatre was due to wound dehiscence or necrosis of flap in 11.7% of patients and urinary leak in 4% [15]. Brown et al. detail a 62% median complication rate for their patient cohort and with 40% classed as grade III/IV CD [15]. Median blood loss was highly variable between studies—Shaikh et al. reported only 800 mL as their median whereas Brown et al. reported 4.2-L average loss within a larger cohort which is inclusive of nerve resection [9, 15]. Sutton et al. reported statistically significant rates of long-term development of empty pelvis syndrome in patients who underwent nerve resection during their radical pelvic exenteration surgery (*p* = 0.03) [17].

### Oncological outcomes

All six included studies reported R0 resection rates and oncological outcomes; however, only three reported any data specifically in the setting of the nerve resection subcohort as outlined (Table 5).

Brown et al. describe R0 rates of 65% for colorectal cancer overall and 68% in locally recurrent colorectal cancer, with no statistical difference between the rates achieved in partial or complete sciatic nerve resections (58 and 66%, respectively, *p* = 0.56 for any significant difference) across a cohort of 39 curative colorectal cancer resections involving nerve resection [15]. This study also reported median survival rates of 41 months for colorectal cancer patients (95% CI (21.9–60.1)) and no difference in survival for patients with or without nerve resection (*p* = 0.684 for any significant difference) [15]. R0 resection was associated with higher rates of 2-year recurrence-free survival (89% vs 13%, *p* < 0.001) with no difference found between partial or complete nerve resection [15]. Brown et al. note similar rates of 1, 3 and 5-year survival for locally advanced vs recurrent colorectal cancer managed with radical resection (92%, 54% and 43% for primary vs 93%, 52% and 41% for recurrent) [15]. Ten of 36 (28%) patients within the colorectal cancer subcohort for whom data was available developed local recurrence following their resection [15].

Solomon et al. have not specifically reported R0 status or survival in the setting of nerve resection but note no overall or disease-free survival disadvantage from sciatic nerve excision (*p* = 0.78, *p* = 0.773, respectively) [16]. Shaikh et al. achieved 100% R0 rates in their six-patient cohort via the extended lateral pelvic sidewall excision (ELSiE) approach; however, they did not describe overall survival and recurrence rates in their data set [9].

### Functional outcomes

#### Function

Two studies reported outcomes pertaining to quality of life as pertaining to physical and mental function. Makker et al. noted that pelvic exenteration patients as a general cohort had reduced physical function as measured with the 6-min walk test as compared to the general population (*p* < 0.001) [18]. Postoperatively, a further decrease was noted at day 10 (*p* < 0.001), and this improved by the time of discharge but was still significantly decreased compared to the preoperative (*p* = 0.03) [15]. There was no significant difference between patients who had undergone major pelvic nerve resection and those that had not (*p* < 0.05) [15]. These data points also held true for the five times sit-to-stand test, with a reduced baseline compared to the general population (*p* < 0.001) which reduced further at day 10 postoperatively (*p* < 0.001) and improved but remained decreased at discharge (*p* = 0.05) [15].

Brown et al. collected prospective postoperative quality of life data with a functional assessment of cancer therapy questionnaire specific to colorectal cancer (FACT-C) for patients who had undergone major nerve resection [15]. Functional data from the immediate postoperative period was also collected. Ninety-six percent of patients could mobilise independently (61% with a walking aid) at discharge in complete sciatic resection, as well as 92% of patients at discharge following partial sciatic resection [15]. Of note, 52% of patients had neuropathic pain of some nature at discharge following complete sciatic nerve resection and 65% of patients who underwent partial sciatic nerve resection suffered the same [15]. Questionnaire data points were collected at preoperative baseline and at 6 and 12 months postoperatively. Forty patients were enrolled in this aspect of the study initially, with 26 patients completing follow-up at 6 months and 20 at 12 months postoperatively. A statistically significant decrease in physical component scales of the FACT-C was noted at 6 months postoperatively for patients who had undergone nerve resection (*p* = 0.041)—these scores had returned to a statistically significant baseline at 12 months postoperatively (*p* = 0.163 for difference between baseline and 12-month score) [15]. No significant differences were noted in the mental component scale or in overall FACT-C scoring at discharge, at 6 months postoperatively or at 12 months postoperatively, and no difference was noted between complete or partial sciatic nerve resection with regards to quality of life scoring [15].

### Pain

One study explored the impact of nerve resection in pelvic exenteration patient cohorts on postoperative pain in a cohort of 42 patients. Lim et al. detailed that 10 of 42 (23%, *p* = 0.223) patients in this cohort were on preoperative opiate pain management, not considered to be a statistically significant deviation from the overall exenteration cohort without resection [7]. Nerve resection was not associated with worse postoperative verbal numerical rating scale (VNRS) pain scores in a statistically significant fashion for any nerve resection subtype (Table 7) [7].

**Table 7:** Pain-related outcomes

Study	Preoperative pain	Preoperative opioids	Postoperative pain—sciatic nerve excision	Postoperative pain—gluteal nerve excision	Postoperative pain—femoral nerve excision	Postoperative pain—obturator nerve excision	Postoperative opioids
Lim et al. [7]	*N* = 30 (30.3%) had significant pain managed by using opiates, with 12 (40.4%) managed on more than 1 opiate	*N* = 30 (30.3%) had significant pain managed by using opiates, with 12 (40.4) managed on more than 1 opiate	None/mild (*n* = 10), moderate (*n* = 4), severe (*n* = 0)—sciatic nerve excision not associated with worse postoperative pain in chi-squared test (*p* = 0.184)	None/mild (*n* = 10), moderate (*n* = 12), severe (*n* = 1) (*p* = 0.318)	None/mild (*n* = 1), moderate (*n* = 0), severe (*n* = 0) (*p* = 0.618)	None/mild (*n* = 7), moderate (*n* = 9), severe (*n* = 3) (*p* = 0.252)	67% of patients required opiates upon discharge; 41% also discharged on antineuropathic agent. This increased 60.5% vs 27.8% if already on preoperative opiates (*p* = 0.001)

Irrespective of any requirement for nerve resection intraoperatively, 41% of patients were discharged on an antineuropathic agent from a preoperative baseline of 11%. Preoperative opiate prescription increased this percentage in a statistically significant fashion (60.5% vs 27.8%, *p* = 0.001) [7]. Table 8 and 9.

## Discussion

This review demonstrates that *en bloc* resection of nerves for colon and rectal cancer remains understudied with only six applicable studies identified. The limited data from these studies however is encouraging. R0 margins are technically feasible and achieved in a majority of patients with locally recurrent rectal cancer undergoing curative resection, with no overall or recurrence-free survival disadvantage despite the challenge posed by resection of major pelvic nerves [15, 16]. The oncological success is balanced against the relatively morbid nature of an aggressive resective approach [17, 19]. With regard to quality of life, radical resection appears to convey at least a mild impact on physical quality of life but with most patients nonetheless ambulating independently at discharge despite resection of major pelvic nerves [15, 18]. Additionally, radical resection involving pelvic nerves appears to improve postoperative pain for patients that undergo this procedure [7]. Major pelvic nerve involvement no longer represents a contraindication to curative resection.

Surgical outcomes for the six papers were highly variable in almost all considered areas and did not stratify for outcomes in major pelvic nerve excision except for the data reported by Brown et al. This study was achieved in a high-volume centre with high-volume surgeons and allowed for postoperative review daily by a complex pelvic and advanced gastrointestinal malignancy surgery experienced physiotherapist, a personalised exercise programme and occupational therapy. Even in this specialised setting, an overall complication rate of 63% and return to theatre rate of 27.9% highlight the challenging postoperative course that radical pelvic sidewall resection with involvement of major pelvic nerves can offer [15]. The need for the development of clear enhanced recovery after surgery (ERAS) protocols is established to improve outcomes in general pelvic exenterative surgeries and should be specialised further within centres performing nerve resection to achieve optimal postoperative outcomes [20]. The other papers considered reported outcomes broadly consistent with existing pelvic exenteration data but with minimal substratification for major nerve resection [4, 21].

With regard to oncological outcomes, R0 margins were achieved in 65% of locally advanced and 68% of locally recurrent colorectal cancers within the largest cohort reported specifically involving nerve resection [15]. These outcomes are comparable to general pelvic exenteration rates of complete oncologic resection and further supported by Solomon et al. noting no survival or recurrence-free disadvantage conveyed by the need for pelvic nerve resection [16, 22]. Given the technical and anatomical challenges posed by radical colorectal resections, centralisation to high-volume centres is the best evidence-based approach to maximise exposure for a select number of specialised quaternary units to achieve complete pathological clearance for as many patients as possible where major nerve resection is required [9, 10, 23, 24]. Centralisation allows for rigorous prehabilitation protocols to be established and practised for patients preoperatively, improving their likelihood of optimal postoperative outcomes [25]. Multidisciplinary review in specialised centres is key to best preoperative planning and subsequent postoperative review where positive margins are achieved to improve interdisciplinary communication, radiological review and technical planning for subsequent cases [26]. All of these protocols underscore the critical importance of appropriate patient selection; given the high rates of postoperative complications encountered in studies to date as outlined in this review, these processes effectively allow surgeons to ensure patients are in an appropriate preoperative state for a successful outcome. Larger studies in specialised centres are needed to enhance the overall evidence base for radical resection involving major pelvic nerves and stratify preoperative, intraoperative and postoperative factors that may contribute to the ability to achieve R0 margins.

As higher and wider curative resections involving major pelvic nerves become technically feasible, achieving the best possible quality of life postoperatively should be a key feature of the developing approach to radical resection going forward. The data elucidated with regard to functional outcomes in this study is sparse but illustrates several key points for further research going forward. Brown et al. noted a statistically significant decrease in physical quality of life as assessed with a FACT-C score at 6 months—this was persistent in terms of median values but had recovered at 12 months such that any difference was no longer statistically significant (*p* = 0.163). Importantly, the 50% loss-to-follow-up rate (40 patients to 20) seen in this subsection of the study is problematic and introduces the potential for significant bias [15]. Encouragingly, in excess of 90% of patients could ambulate independently either with or without gait aids at discharge [15]. This suggests that nerve resection in and of itself may not contribute significantly to any persistent postoperative physical disability where the appropriate aids and orthotics are offered beyond the acknowledged physical disability burden conveyed by pelvic exenteration generally in previous quality of life studies [27]. The study by Makker et al. noting no differential in 6-min walk test or five times sit-to-stand test in the general pelvic exenteration cohort as compared to the cohort undergoing nerve resection further supports this theory [18]. More focused and higher-powered prospective studies examining quality of life in major pelvic nerve resection are required to further explore outcomes in this setting and interventions that may ameliorate any deficits conveyed by resection.

Postoperative pain can have a significant negative impact on patients’ quality of life following radical resectional surgery [28]. The findings by Lim et al. that resection involving major pelvic nerve resection was not associated with worse postoperative pain outcomes are significant [7]. The authors also postulate that preoperative infiltration of the nerve with tumour may thus mean that nerve resection does not contribute significantly to pain compared to exenteration as a whole. Nonetheless, 67% of pelvic exenteration patients are discharged on postoperative opiates and 41% on neuropathic pain agents, suggesting that there may be an underreported or under-recognised burden of neuropathic pain not elucidated in this single study. This was also recognised within the Brown et al. cohort, with half of patients discharged on neuropathic pain agents [15]. Previous systematic reviews on postoperative pain in pelvic exenteration have suggested studies utilise tailored neuropathic pain questionnaires to follow patients longitudinally with regards to this symptom burden [28, 29]. Such a study would be of significant utility in elucidating the role of nerve resection in contributing to postoperative neuropathic pain and thus considering possible interventions for ameliorating this in the perioperative and intraoperative phase.

Limitations to this systematic review include the low study numbers involved and the between patients’ tumour pathologies, outcomes and lack of stratification between pelvic exenteration with and without nerve resection and for colorectal tumour type specifically in many instances. Additionally, five of the six papers draw from a single centre’s prospective studies, and as such, study results are subject to the possibility of selection bias resulting from the possibility that individual patients have been included across multiple studies. The inclusion of 178 separate major nerve resections within the study by Sutton et al. nonetheless suggests that this study still represents the broadest possible summary of major nerve resections described in the literature to date and minimises this risk as much as feasible within the limits of a systematic review [18]. Only two papers reported on long-term quality of life impacts of this surgery but reported this via different measures and both within the first year of the postoperative course. The only paper that solely conducted a study based on the patient cohort who underwent nerve resection alongside their PE had a non-disclosed loss to follow-up that introduces the potential for underreporting or bias. The outcomes reported for postoperative pain were also relatively limited in scope and did not consider neuropathic pain specifically. The retrospective nature of all studies considered also confers a level of unavoidable bias which future prospective studies could limit.

In the short term, this study has significant potential to provide an objective basis for a valuable preoperative counselling resource for exenterative surgeons outlining the potential benefits and risks of radical resection involving major nerve resection. Data summarising the favourable oncological risk profile and lack of persistent functional deficits at 12 months can be incorporated alongside salient task-based information regarding the steps involved in postoperative rehabilitation and the associated timelines to restorative care in this setting. Validation of such a resource in a prospective study would be highly useful to both patients and clinicians.

Future direction for research relies on multicentre international collaboration given the low case numbers per individual unit. High-volume exenteration units should collaborate in examining functional outcomes and in developing strategies to mitigate morbidity and maximise functional outcome postoperatively. Interventions to improve outcomes could include the formation of specialised enhanced recovery after surgery guidelines centred around pain management, exercise, nutrition, counselling and targeted gait aids to maximise mobility. Targeted studies to explore the prevalence and significance of neuropathic pain would be of significant benefit, as well as intraoperative trials using local anaesthetic applied to the nerve for neuropathic pain prevention. The role of minimally invasive surgery techniques in centres with appropriate expertise to limit morbidity and blood loss is increasing and has been described previously in the setting of major nerve resection for locally recurrent colorectal cancer [30]. The potential for superior surgical and oncological outcomes in this setting would be of note to establish in a larger cohort given the significant established morbidity of open resection.

## Conclusion

The existing outcomes for resection of locally advanced and recurrent colorectal cancer involving the need for major nerve resection are encouraging. R0 resections remain the most prognostic factor for overall survival and recurrence-free survival, and these can be readily achieved in at least two-thirds of patients even where major pelvic nerves are involved. The technically complex and radical nature of these resections suggests that centralisation to high-volume centres results in the best postoperative outcomes secondary to capacity for extensive prehabilitation, multidisciplinary review and technical expertise. Functional outcome data is lacking in contemporary literature with regards to major pelvic nerve resection but suggests that the majority of patients will be able to ambulate at discharge despite major pelvic nerve resection and without any additional postoperative pain burden conveyed by the nerve resection specifically. Establishing this oncological and function data in a review setting allows for the development of preoperative education aids that may be of significant utility to clinicians and patients considering radical resection. Further studies will explore in greater detail any potential functional deficits suffered by long-term survivors, any potential for unrecognised neuropathic pain burden and the potential role of minimally invasive surgery to improve surgical and oncological outcomes.

## Data Availability

The datasets generated during and/or analysed during the current study are available from the corresponding author on reasonable request.

## Appendix 1. Search strategy

OVID Medline + Embase—1044 records identified

1. colorectal*.mp. 557882
2. rectal*.mp. 324,045
3. exp Pelvic Neoplasms/ 17,599
4. exp Rectal Neoplasms/ 135,435
5. surg*.mp. 8,970,608
6. pelv*.mp. 532,439
7. radical resection.mp. 24,088
8. pelvic nerves.mp. 1288
9. exp sciatic nerve/ 63,515
10. exp femoral nerve/ 9533
11. exp obturator nerve/ 2741
12. aortoiliac.mp. 8065
13. nerve.tw. 983,323
14. 1 or 2 or 3 or 4 868,122
15. 8 or 9 or 10 or 11 or 12 or 13 1,006,648
16. 6 or 7 555,127
17. 5 and 14 and 15 and 16 2301
18. remove duplicates from 17 1646
19. limit 18 to english language 1432
20. limit 19 to yr = “2010 -Current” 1044

Web of Science—719 records identified

(ALL = (colorectal*) OR ALL = (rectal*) OR ALL = (exp pelvic neoplasms) OR ALL = (exp rectal neoplasms)) AND ALL = (surg*) AND (ALL = (pelv*) OR ALL = (radical resection)) AND (ALL = (pelvic nerves) OR ALL = (exp sciatic nerve) OR ALL = (exp femoral nerve) OR ALL = (exp obturator nerve) OR ALL = (aortoiliac) OR ALL = (nerve))

## Appendix 2. Risk of bias assessment for included studies

**Table 8:** Newcastle–Ottawa Quality Assessment Scale for the included studies

No	Criterion	Question	Score (* = 1, no * = 0)					
Studies			Shaikh et al. [9]	Solomon et al. [16]	Lim et al. [7]	Makker et al. [18]	Brown et al. [15]	Sutton et al. [17]
Selection								
1	Adequacy of case definition	*a) yes, with independent validation ** *b) yes, e.g., record linkage or based on self-reports* *c) no description*	1	0	1	0	1	1
2	Representativeness of cases	*a) consecutive or obviously representative series of cases ** *b) potential for selection biases or not stated*	1	1	1	0	1	1
3	Selection of controls	*a) community controls ** *b) hospital controls* *c) no description*	0	0	0	0	0	0
4	Definition of controls	*a) no history of disease (endpoint) ** *b) no description of source*	1	1	1	1	1	1
Comparability								
1	Comparability of cases and controls on basis of design or analysis	*a) study controls for _______________ (Select the most important factor.) ** *b) study controls for any additional factor **	Nerve resection via ELSiE	Morbidity and oncological outcomes	Perioperative pain	Physical function	Survival, function and QoL	Empty pelvis syndrome
Exposure								
1	Ascertainment of exposure	*a) secure record (e.g. surgical records) ** *b) structured interview where blind to case/control status ** *c) interview not blinded to case/control status* *d) written self-report or medical record only* *e) no description*	1	1	1	0	1	1
2	Same method of ascertainment for cases and controls	*a) yes ** *b) no*	1	1	1	1	1	1
3	Non-response rate	*a) same rate for both groups ** *b) non respondents described* *c) rate different and no designation*	1	1	0	0	0	1
Score:			7	6	6	3	6	7

## Appendix 3.

**Table 9:** PRISMA checklist

Section and topic	Item #	Checklist item	Location where item is reported (page #)
**Title**			
Title	1	Identify the report as a systematic review	p.1
**Abstract**			
Abstract	2	See the PRISMA 2020 for Abstracts checklist	p.2
**Introduction**			
Rationale	3	Describe the rationale for the review in the context of existing knowledge	p.3–4
Objectives	4	Provide an explicit statement of the objective(s) or question(s) the review addresses	p.4
**Methods**			
Eligibility criteria	5	Specify the inclusion and exclusion criteria for the review and how studies were grouped for the syntheses	p.14
Information sources	6	Specify all databases, registers, websites, organisations, reference lists and other sources searched or consulted to identify studies. Specify the date when each source was last searched or consulted	p.4
Search strategy	7	Present the full search strategies for all databases, registers and websites, including any filters and limits used	p.23
Selection process	8	Specify the methods used to decide whether a study met the inclusion criteria of the review, including how many reviewers screened each record and each report retrieved, whether they worked independently, and if applicable, details of automation tools used in the process	p.4–5
Data collection process	9	Specify the methods used to collect data from reports, including how many reviewers collected data from each report, whether they worked independently, any processes for obtaining or confirming data from study investigators, and if applicable, details of automation tools used in the process	p.4–5
Data items	10a	List and define all outcomes for which data were sought. Specify whether all results that were compatible with each outcome domain in each study were sought (e.g. for all measures, time points, analyses), and if not, the methods used to decide which results to collect	p.5
	10b	List and define all other variables for which data were sought (e.g. participant and intervention characteristics, funding sources). Describe any assumptions made about any missing or unclear information	p.5
Study risk of bias assessment	11	Specify the methods used to assess risk of bias in the included studies, including details of the tool(s) used, how many reviewers assessed each study and whether they worked independently, and if applicable, details of automation tools used in the process	p.5
Effect measures	12	Specify for each outcome the effect measure(s) (e.g. risk ratio, mean difference) used in the synthesis or presentation of results	p.6–9
Synthesis methods	13a	Describe the processes used to decide which studies were eligible for each synthesis (e.g. tabulating the study intervention characteristics and comparing against the planned groups for each synthesis (item #5))	p.14
	13b	Describe any methods required to prepare the data for presentation or synthesis, such as handling of missing summary statistics, or data conversions	p.6
	13c	Describe any methods used to tabulate or visually display results of individual studies and syntheses	p.6
	13d	Describe any methods used to synthesize results and provide a rationale for the choice(s). If meta-analysis was performed, describe the model(s), method(s) to identify the presence and extent of statistical heterogeneity, and software package(s) used	p.7–9
	13e	Describe any methods used to explore possible causes of heterogeneity among study results (e.g. subgroup analysis, meta-regression)	p.12
	13f	Describe any sensitivity analyses conducted to assess robustness of the synthesized results	Not performed
Reporting bias assessment	14	Describe any methods used to assess risk of bias due to missing results in a synthesis (arising from reporting biases)	p.6
Certainty assessment	15	Describe any methods used to assess certainty (or confidence) in the body of evidence for an outcome	p.12
**Results**			
Study selection	16a	Describe the results of the search and selection process, from the number of records identified in the search to the number of studies included in the review, ideally using a flow diagram	p.6
	16b	Cite studies that might appear to meet the inclusion criteria, but which were excluded, and explain why they were excluded	p.6
Study characteristics	17	Cite each included study and present its characteristics	p.7–9, p.28–29
Risk of bias in studies	18	Present assessments of risk of bias for each included study	p.24–25
Results of individual studies	19	For all outcomes, present, for each study: (a) summary statistics for each group (where appropriate) and (b) an effect estimate and its precision (e.g. confidence/credible interval), ideally using structured tables or plots	p.7–9, p.15–22
Results of syntheses	20a	For each synthesis, briefly summarise the characteristics and risk of bias among contributing studies	p.7–9, p.24–25
	20b	Present results of all statistical syntheses conducted. If meta-analysis was done, present for each the summary estimate and its precision (e.g. confidence/credible interval) and measures of statistical heterogeneity. If comparing groups, describe the direction of the effect	p.15–22
	20c	Present results of all investigations of possible causes of heterogeneity among study results	p.12
	20d	Present results of all sensitivity analyses conducted to assess the robustness of the synthesized results	Not performed
Reporting biases	21	Present assessments of risk of bias due to missing results (arising from reporting biases) for each synthesis assessed	p.24–25
Certainty of evidence	22	Present assessments of certainty (or confidence) in the body of evidence for each outcome assessed	Not performed
**Discussion**			
Discussion	23a	Provide a general interpretation of the results in the context of other evidence	p.10–12
	23b	Discuss any limitations of the evidence included in the review	p.12
	23c	Discuss any limitations of the review processes used	p.12
	23d	Discuss implications of the results for practice, policy, and future research	p.12–13
**Other information**			
Registration and protocol	24a	Provide registration information for the review, including register name and registration number, or state that the review was not registered	p.4
	24b	Indicate where the review protocol can be accessed, or state that a protocol was not prepared	p.4
	24c	Describe and explain any amendments to information provided at registration or in the protocol	N/A
Support	25	Describe sources of financial or non-financial support for the review, and the role of the funders or sponsors in the review	p.1
Competing interests	26	Declare any competing interests of review authors	p.1
Availability of data, code and other materials	27	Report which of the following are publicly available and where they can be found: template data collection forms; data extracted from included studies; data used for all analyses; analytic code; any other materials used in the review	p.3

## References

[1] Yang TX, Morris DL, Chua TC (2013) Pelvic exenteration for rectal cancer. Dis Colon Rectum 56:519–531. 10.1097/DCR.0b013e31827a7868 23478621 10.1097/DCR.0b013e31827a7868

[2] Kim J (2012) Pelvic Exenteration: Surgical Approaches. J Korean Soc Coloproctol 28:286. 10.3393/jksc.2012.28.6.286 23346506 10.3393/jksc.2012.28.6.286 PMC3548142

[3] Yun J-A, Huh JW, Kim HC et al (2016) Local recurrence after curative resection for rectal carcinoma. Medicine 95:e3942. 10.1097/MD.0000000000003942 27399067 10.1097/MD.0000000000003942 PMC5058796

[4] Collaborative PelvEx (2019) Surgical and survival outcomes following pelvic exenteration for locally advanced primary rectal cancer. Ann Surg 269:315–321. 10.1097/SLA.0000000000002528 28938268 10.1097/SLA.0000000000002528

[5] Lau YC, Brown KGM, Lee P (2019) Pelvic exenteration for locally advanced and recurrent rectal cancer—how much more? J Gastrointest Oncol 10:1207–1214. 10.21037/jgo.2019.01.21 31949941 10.21037/jgo.2019.01.21 PMC6954998

[6] Austin KKS, Solomon MJ (2009) Pelvic exenteration with en bloc iliac vessel resection for lateral pelvic wall involvement. Dis Colon Rectum 52:1223–1233. 10.1007/DCR.0b013e3181a73f48 19571697 10.1007/DCR.0b013e3181a73f48

[7] Lim JS-Y, Koh CE, Liu H et al (2018) The price we pay for radical curative pelvic exenterations: prevalence and management of pain. Dis Colon Rectum 61:314–319. 10.1097/DCR.0000000000001013 29420427 10.1097/DCR.0000000000001013

[8] Brown KGM, Solomon MJ, Koh CE (2017) Pelvic exenteration surgery: the evolution of radical surgical techniques for advanced and recurrent pelvic malignancy. Dis Colon Rectum 60:745–754. 10.1097/DCR.0000000000000839 28594725 10.1097/DCR.0000000000000839

[9] Shaikh I, Aston W, Hellawell G et al (2014) Extended lateral pelvic sidewall excision (ELSiE): an approach to optimize complete resection rates in locally advanced or recurrent anorectal cancer involving the pelvic sidewall. Tech Coloproctol 18:1161–1168. 10.1007/s10151-014-1234-9 25380742 10.1007/s10151-014-1234-9

[10] Warrier SK, Heriot AG, Lynch AC (2016) Surgery for locally recurrent rectal cancer: tips, tricks, and pitfalls. Clin Colon Rectal Surg 29:114–122. 10.1055/s-0036-1580723 27247536 10.1055/s-0036-1580723 PMC4882182

[11] Koh CE, Solomon MJ, Brown KG et al (2017) The evolution of pelvic exenteration practice at a single center: lessons learned from over 500 cases. Dis Colon Rectum 60:627–635. 10.1097/DCR.0000000000000825 28481857 10.1097/DCR.0000000000000825

[12] Rahbari NN, Ulrich AB, Bruckner T et al (2011) Surgery for locally recurrent rectal cancer in the era of total mesorectal excision. Ann Surg 253:522–533. 10.1097/SLA.0b013e3182096d4f 21209587 10.1097/SLA.0b013e3182096d4f

[13] Collaborative PelvEx (2022) Contemporary management of locally advanced and recurrent rectal cancer: views from the PelvEx collaborative. Cancers (Basel) 14:1161. 10.3390/cancers14051161 35267469 10.3390/cancers14051161 PMC8909015

[14] Steffens D, Solomon MJ, Young JM et al (2018) Cohort study of long-term survival and quality of life following pelvic exenteration. BJS Open 2:328–335. 10.1002/bjs5.75 30263984 10.1002/bjs5.75 PMC6156168

[15] Brown KGM, Solomon MJ, Lau YC et al (2021) Sciatic and femoral nerve resection during extended radical surgery for advanced pelvic tumours. Ann Surg 273:982–988. 10.1097/SLA.0000000000003390 31188210 10.1097/SLA.0000000000003390

[16] Solomon MJ, Brown KGM, Koh CE et al (2015) Lateral pelvic compartment excision during pelvic exenteration. Br J Surg 102:1710–1717. 10.1002/bjs.9915 26694992 10.1002/bjs.9915

[17] Ouzzani M, Hammady H, Fedorowicz Z, Elmagarmid A (2016) Rayyan—a web and mobile app for systematic reviews. Syst Rev 5:210. 10.1186/s13643-016-0384-4 27919275 10.1186/s13643-016-0384-4 PMC5139140

[18] Sutton PA, Brown KGM, Ebrahimi N et al (2022) Long-term surgical complications following pelvic exenteration: operative management of the empty pelvis syndrome. Colorectal Dis 24:1491–1497. 10.1111/codi.16238 35766998 10.1111/codi.16238

[19] Makker PGS, Koh CE, Solomon MJ et al (2021) Functional outcomes following pelvic exenteration: results from a prospective cohort study. Colorectal Dis 23:2647–2658. 10.1111/codi.15834 34346149 10.1111/codi.15834

[20] Venchiarutti RL, Solomon MJ, Koh CE et al (2019) Pushing the boundaries of pelvic exenteration by maintaining survival at the cost of morbidity. Br J Surg 106:1393–1403. 10.1002/bjs.11203 31282571 10.1002/bjs.11203

[21] Harji D, Mauriac P, Bouyer B et al (2021) The feasibility of implementing an enhanced recovery programme in patients undergoing pelvic exenteration. Eur J Surg Oncol 47:3194–3201. 10.1016/j.ejso.2021.07.013 34736803 10.1016/j.ejso.2021.07.013

[22] Platt E, Dovell G, Smolarek S (2018) Systematic review of outcomes following pelvic exenteration for the treatment of primary and recurrent locally advanced rectal cancer. Tech Coloproctol 22:835–845. 10.1007/s10151-018-1883-1 30506497 10.1007/s10151-018-1883-1

[23] Warren OJ, Solomon MJ (2015) R0 resection, not surgical technique, is the key consideration in pelvic exenteration surgery. Tech Coloproctol 19:117–118. 10.1007/s10151-014-1256-3 25544128 10.1007/s10151-014-1256-3

[24] Althumairi AA, Canner JK, Gorin MA et al (2016) Reduction of costs for pelvic exenteration performed by high volume surgeons: analysis of the maryland health service cost review commission database. Am Surg 82:46–52 26802857

[25] van Gijn W, Gooiker GA, Wouters MWJM et al (2010) Volume and outcome in colorectal cancer surgery. Eur J Surg Oncol 36(Suppl 1):S55-63. 10.1016/j.ejso.2010.06.027 20615649 10.1016/j.ejso.2010.06.027

[26] Minnella EM, Bousquet-Dion G, Awasthi R et al (2017) Multimodal prehabilitation improves functional capacity before and after colorectal surgery for cancer: a five-year research experience. Acta Oncol (Madr) 56:295–300. 10.1080/0284186X.2016.1268268 10.1080/0284186X.2016.1268268 28079430

[27] van Ramshorst GH, O’Shannassy S, Brown WE et al (2018) A qualitative study of the development of a multidisciplinary case conference review methodology to reduce involved margins in pelvic exenteration surgery for recurrent rectal cancer. Colorectal Dis 20:1004–1013. 10.1111/codi.14311 29920909 10.1111/codi.14311

[28] Austin KKS, Young JM, Solomon MJ (2010) Quality of life of survivors after pelvic exenteration for rectal cancer. Dis Colon Rectum 53:1121–1126. 10.1007/DCR.0b013e3181e10c46 20628274 10.1007/DCR.0b013e3181e10c46

[29] Johnstone CSH, Roberts D, Mathieson S et al (2023) Pain, pain management and related outcomes following pelvic exenteration surgery: a systematic review. Colorectal Dis 25:562–572. 10.1111/codi.16462 36572393 10.1111/codi.16462

[30] Krause SJ, Backonja M-M (2003) Development of a neuropathic pain questionnaire. Clin J Pain 19:306–314. 10.1097/00002508-200309000-00004 12966256 10.1097/00002508-200309000-00004

[31] Rajkomar A, Larach T, Mohan H et al (2022) Video vignette: robotic pelvic sidewall resection with en bloc sciatic nerve excision. Colorectal Dis. 10.1111/codi.16401 36324061 10.1111/codi.16401

